# The Treatment of Gall-Stones at Leeds General Infirmary

**Published:** 1893-05-27

**Authors:** 


### THE TREATMENT OF GALL-STONES AT LEEDS GENERAL INFIRMARY.

The report on "The Surgical Treatment of Gall-stones," by Professor Mayo-Robson, which appeared in our issue of May 13th, should have been accredited to Mr. B. L. Whitehead, M.B., instead of to Professor Mayo-Robson himself, to whom, however, we are indebted for kindly revising the article.

